# Supplementary material for: Influenza A Virus Infection Reactivates Human Endogenous Retroviruses Associated with Modulation of Antiviral Immunity
Source: Viruses. 2022 Jul 21;14(7):1591. doi: 10.3390/v14071591 (PMC9320126; doi:10.3390/v14071591)
Supplement: Supplementary file 1 [file viruses-14-01591-s001.zip › MotifAnalysis.zip!/MotifOutput_SC35MdNS1/homerResults.html]

MotifOutput// - Homer de novo Motif Results


# Homer *de novo* Motif Results (MotifOutput//)

Known Motif Enrichment Results  
Gene Ontology Enrichment Results  
If Homer is having trouble matching a motif to a known motif, try copy/pasting the matrix file into
STAMP  
More information on motif finding results: HOMER
| Description of Results
| Tips
  
Total target sequences = 77  
Total background sequences = 45412  
\* - possible false positive  

|  |  |  |  |  |  |  |  |  |
| --- | --- | --- | --- | --- | --- | --- | --- | --- |
| Rank | Motif | P-value | log P-pvalue | % of Targets | % of Background | STD(Bg STD) | Best Match/Details | Motif File |
| 1 | A C G T G A T C A C G T C G T A A C T G A G T C A C G T A G T C C G T A C T G A A C T G A C T G | 1e-78 | -1.817e+02 | 50.65% | 0.26% | 57.2bp (110.9bp) | ZBTB6/MA1581.1/Jaspar(0.715) More Information \| Similar Motifs Found | motif file (matrix) |
| 2 | A G T C C G T A G T A C A G T C G T C A C T G A A G C T A T G C C T G A C T A G A G T C C T G A | 1e-71 | -1.651e+02 | 53.25% | 0.52% | 56.0bp (97.0bp) | NFYA/MA0060.3/Jaspar(0.736) More Information \| Similar Motifs Found | motif file (matrix) |
| 3 | A G T C A C G T C T A G A C G T C T G A A C G T G A T C A C G T C G T A A C T G A G T C A C G T | 1e-70 | -1.617e+02 | 49.35% | 0.37% | 53.3bp (107.0bp) | MEF2B/MA0660.1/Jaspar(0.650) More Information \| Similar Motifs Found | motif file (matrix) |
| 4 | G A T C C G T A C T G A A C T G C T A G C G A T A G C T A C G T A C T G A C G T C G T A C G T A | 1e-68 | -1.588e+02 | 51.95% | 0.53% | 53.3bp (96.5bp) | SRY/MA0084.1/Jaspar(0.643) More Information \| Similar Motifs Found | motif file (matrix) |
| 5 | A G T C A C G T A C G T C T A G A T C G C G T A T A C G G C T A C A T G G A T C A G T C A G C T | 1e-57 | -1.329e+02 | 41.56% | 0.33% | 50.0bp (88.1bp) | Nkx2-5(var.2)/MA0503.1/Jaspar(0.587) More Information \| Similar Motifs Found | motif file (matrix) |
| 6 | C G T A A G T C A C G T A G T C A C G T A C T G A C G T C G T A A C G T A G T C | 1e-55 | -1.271e+02 | 44.16% | 0.54% | 43.9bp (82.2bp) | Dmrt1/MA1603.1/Jaspar(0.768) More Information \| Similar Motifs Found | motif file (matrix) |
| 7 | C T A G C G T A C T A G C G T A C G T A A G T C A G T C A C G T A C G T A C G T C T G A A C G T | 1e-51 | -1.184e+02 | 35.06% | 0.22% | 52.0bp (127.7bp) | OVOL1/MA1544.1/Jaspar(0.700) More Information \| Similar Motifs Found | motif file (matrix) |
| 8 | T A G C A C G T A G T C A C G T A C T G C A T G A C G T A C T G C T A G A C T G A C T G G T C A | 1e-49 | -1.142e+02 | 36.36% | 0.31% | 47.1bp (92.7bp) | KLF4/MA0039.4/Jaspar(0.629) More Information \| Similar Motifs Found | motif file (matrix) |
| 9 | C T A G A C G T A C T G C T A G C T A G A C T G G C T A A G T C A C G T A G C T | 1e-35 | -8.270e+01 | 33.77% | 0.69% | 51.6bp (82.9bp) | MZF1/MA0056.2/Jaspar(0.752) More Information \| Similar Motifs Found | motif file (matrix) |
| 10 | A G T C A C G T C G T A A C T G A G T C A C G T A C T G A G T C | 1e-30 | -6.942e+01 | 29.87% | 0.72% | 46.2bp (88.2bp) | BHLHE22(var.2)/MA1635.1/Jaspar(0.698) More Information \| Similar Motifs Found | motif file (matrix) |
| 11 | A C T G A C T G A C T G A G C T A C G T A G C T T A G C A C G T A C T G C G T A | 1e-19 | -4.414e+01 | 19.48% | 0.50% | 47.2bp (85.0bp) | OSR2/MA1646.1/Jaspar(0.672) More Information \| Similar Motifs Found | motif file (matrix) |
| 12 | A G T C A C G T G T A C C G T A C A T G A C T G A C T G A C G T C G A T G T C A C G T A A C G T | 1e-17 | -4.088e+01 | 10.39% | 0.03% | 50.7bp (45.7bp) | Nkx6.1(Homeobox)/Islet-Nkx6.1-ChIP-Seq(GSE40975)/Homer(0.644) More Information \| Similar Motifs Found | motif file (matrix) |
| 13 | A G T C C G T A A G C T A G T C A G T C C T A G C G T A A C T G A C T G C G T A C G T A A C G T | 1e-17 | -4.086e+01 | 7.79% | 0.00% | 47.5bp (8.9bp) | PB0006.1\_Bcl6b\_1/Jaspar(0.678) More Information \| Similar Motifs Found | motif file (matrix) |
| 14 | G T A C A C T G A C T G C T G A A G T C A G T C C G T A A C T G A C T G C G T A C G T A C G T A | 1e-17 | -3.981e+01 | 9.09% | 0.02% | 46.7bp (45.1bp) | IKZF1/MA1508.1/Jaspar(0.704) More Information \| Similar Motifs Found | motif file (matrix) |
| 15 | A C G T C G T A C G T A A G T C A G T C C G T A A C T G A C T G A C G T C G T A A C G T A C G T | 1e-15 | -3.679e+01 | 6.49% | 0.00% | 41.7bp (0.0bp) | ZEB1(Zf)/PDAC-ZEB1-ChIP-Seq(GSE64557)/Homer(0.656) More Information \| Similar Motifs Found | motif file (matrix) |
| 16 | C G T A A C T G C G T A A G T C A C G T A C G T A C G T A C T G A G T C A C G T A G T C A C G T | 1e-15 | -3.679e+01 | 6.49% | 0.00% | 60.6bp (0.0bp) | LEF1/MA0768.1/Jaspar(0.665) More Information \| Similar Motifs Found | motif file (matrix) |
| 17 | A C G T A C G T A C T G C G T A A G T C A C G T A C G T A G T C A G T C A C G T A G T C A G T C | 1e-15 | -3.679e+01 | 6.49% | 0.00% | 51.8bp (26.9bp) | SPI1/MA0080.5/Jaspar(0.743) More Information \| Similar Motifs Found | motif file (matrix) |
| 18 | T G A C C T A G C G T A A C G T A G T C A G T C C G T A A G T C A C G T C T A G A C T G C T A G | 1e-14 | -3.292e+01 | 10.39% | 0.09% | 52.5bp (76.0bp) | ZNF354C/MA0130.1/Jaspar(0.722) More Information \| Similar Motifs Found | motif file (matrix) |
| 19 | A C G T A G T C A G T C A C G T A G T C C G A T C G A T A C T G A C T G A C G T A G T C A G T C | 1e-14 | -3.256e+01 | 7.79% | 0.02% | 30.0bp (93.8bp) | Sox4(HMG)/proB-Sox4-ChIP-Seq(GSE50066)/Homer(0.562) More Information \| Similar Motifs Found | motif file (matrix) |
| 20 | A G T C A G T C A G T C A C G T A G C T G T C A A C G T A G C T G C T A C T A G A C T G A T C G | 1e-12 | -2.930e+01 | 10.39% | 0.13% | 35.3bp (58.2bp) | CArG(MADS)/PUER-Srf-ChIP-Seq(Sullivan\_et\_al.)/Homer(0.706) More Information \| Similar Motifs Found | motif file (matrix) |
| 21 | A C T G A C G T A C G T C G T A A C G T A C T G A G T C A G T C A G T C A T C G C G T A C G T A | 1e-12 | -2.875e+01 | 5.19% | 0.00% | 46.8bp (0.0bp) | PB0133.1\_Hic1\_2/Jaspar(0.659) More Information \| Similar Motifs Found | motif file (matrix) |
| 22 \* | A C T G A C G T A G T C T A C G A C G T A G T C G T A C C G T A A C T G A C T G C G T A A G T C | 1e-11 | -2.598e+01 | 5.19% | 0.01% | 27.2bp (32.2bp) | Nr5a2(NR)/mES-Nr5a2-ChIP-Seq(GSE19019)/Homer(0.578) More Information \| Similar Motifs Found | motif file (matrix) |
| 23 \* | C T G A C T A G C A T G C T G A A C G T C T G A C G A T A C T G A C T G C T A G | 1e-10 | -2.466e+01 | 15.58% | 1.00% | 56.0bp (70.0bp) | ETS:RUNX(ETS,Runt)/Jurkat-RUNX1-ChIP-Seq(GSE17954)/Homer(0.825) More Information \| Similar Motifs Found | motif file (matrix) |
| 24 \* | A C T G A T C G A T C G A G T C C T A G A G T C C T A G A C T G A G T C A G T C A G C T A C G T | 1e-10 | -2.436e+01 | 5.19% | 0.01% | 39.1bp (25.3bp) | Zfx/MA0146.2/Jaspar(0.717) More Information \| Similar Motifs Found | motif file (matrix) |
| 25 \* | A G T C A C G T A C T G A G T C C G T A A C T G A G T C A C G T A C G T A T G C C G T A A G T C | 1e-7 | -1.794e+01 | 5.19% | 0.03% | 66.3bp (48.7bp) | Ascl2(bHLH)/ESC-Ascl2-ChIP-Seq(GSE97712)/Homer(0.604) More Information \| Similar Motifs Found | motif file (matrix) |
| 26 \* | A T C G G C T A A C T G A G C T A G C T A C T G A C G T G C T A | 1e-7 | -1.723e+01 | 14.29% | 1.55% | 47.8bp (73.1bp) | MYB/MA0100.3/Jaspar(0.738) More Information \| Similar Motifs Found | motif file (matrix) |
| 27 \* | A C T G A C T G C G T A C G T A A G T C A C T G C G T A C G T A A G T C C G T A C G T A A G T C | 1e-5 | -1.345e+01 | 2.60% | 0.00% | 25.4bp (14.2bp) | SOX12/MA1561.1/Jaspar(0.726) More Information \| Similar Motifs Found | motif file (matrix) |
| 28 \* | A C G T A G T C A C G T A C G T A C G T A C G T A G T C C G T A | 1e-4 | -1.094e+01 | 9.09% | 1.05% | 66.1bp (63.8bp) | PB0022.1\_Gata5\_1/Jaspar(0.724) More Information \| Similar Motifs Found | motif file (matrix) |
